# Needs assessment and Acceptability of a Community First Aid Responder programme to increase Out-of-hospital capacity in Kinshasa, Democratic Republic of Congo: A qualitative study

**DOI:** 10.1016/j.afjem.2024.12.003

**Published:** 2025-01-08

**Authors:** K Diango, J Pigoga, E Mafuta, J Yangongo, L Wallis, C Cunningham, P Hodkinson

**Affiliations:** a Division of Emergency Medicine, Department of Family, Community and Emergency Care, Faculty of Health Sciences, University of Cape Town, South Africa; b Kinshasa School of Public Health, University of Kinshasa. Commune Lemba, Kinshasa, Congo

**Keywords:** Emergency care, First responder, Acceptability, Democratic Republic of Congo

## Abstract

**Objective:**

Despite efforts in recent years to expand the availability of prehospital care in low- and middle-income countries, its availability remains limited in many regions. The World Health Organization advocates the development of layperson first responder programmes as a supportive step in building functioning prehospital systems. This study aimed to identify the need for, and acceptability of, a community first responder programme to increase out-of-hospital capacity in Kinshasa, Democratic Republic of Congo.

**Methods:**

We conducted five focus group discussions using purposive sampling. We included health system planners, emergency care providers, community health volunteers, and community members in both urban and peri‑urban areas. Interviews were recorded and transcribed verbatim, validated, and subjected to inductive content analysis to identify themes and sub-themes.

**Results:**

Several areas of the emergency care system were identified for improvement, starting with the initial response to emergencies in the community. Barriers included planning and governance issues; inadequate resources such as trained staff; cost; transportation issues; and reliance on alternative forms of care. There was a dominant view that sustainably addressing these barriers and building on identified facilitators requires a multi-pronged approach involving government, healthcare, and community members. Perspectives about the acceptability and sustainability of a community first responder programme were largely positive, and numerous actionable recommendations were provided.

**Conclusion:**

A community first responder programme was deemed a useful and acceptable intervention to help increase out-of-hospital emergency care capacity in Kinshasa. Key potential facilitators and barriers to its implementation and sustainability were identified.

## African relevance

• Despite efforts in recent years to expand the availability of prehospital care in low- and middle-income countries in Africa, its availability remains limited in many regions.
• The World Health Organization advocates the development of layperson first responder programmes as a supportive step in building functioning prehospital systems in low-resource settings.
• This study assesses the needs for, and acceptability of, a community-based first responder programme in the Democratic Republic of Congo.
• The study identifies key potential facilitators and barriers to implementation and sustainability of a layperson responder system and provides a useful guide regarding the efforts to improve out-of-hospital emergency care capacity in Africa.

## Introduction

Improving provision of emergency care (EC) in health systems can have a significant impact on morbidity, mortality, and well-being of populations [[1], [2], [3]]. Efficient EC systems that include prehospital and facility-based care are essential for good patients outcomes [2]. Despite the evidence, EC has historically not been prioritised in Africa; only one-third-of countries have prehospital Emergency Medical Services (EMS) systems in place [4] and facility-based EC is often underdeveloped [[5], [6], [7]]. Most health emergencies occur in community spaces and homes, away from health facilities and trained healthcare providers, and community members are often the first on scene. Out-of-Hospital EC (OHEC) capacity broadly relates to the ability of a healthcare system to provide care outside traditional hospital settings [8]. In order to address delays in care at the scenes of emergencies, particularly in low-and-middle income countries (LMICs) with less developed EC systems, immediate bystander intervention could be vital [9]. Substantial evidence has demonstrated the positive impact of laypersons trained in basic EC [10], with improvements across a range of illnesses and injuries, including out-of-hospital cardiac arrest [11], trauma [12], burns [13], and drowning [14].

The World Health Organization (WHO) identified the development of layperson first responder programmes as a fundamental first step in expanding the functionality of prehospital systems [15], and developed the Community First Aid Responder (CFAR) programme to this effect. CFARs are members of the public who receive first aid training and are actively linked into the prehospital system, to help their community by responding to medical emergencies before formal care can be accessed [16]. They can improve access through the integration of EC with community-based health care systems, particularly in poorly-resourced and rural settings [17,18]. Several community-based first responder programmes have been implemented in Africa to varying extents [16,19,20], with increased OHEC capacity and improved access to care as prominent benefits. The Democratic Republic of Congo (DRC) EC system is underdeveloped [[21], [22], [23]] and could benefit from a CFAR programme. As with all health interventions, though, local adaptation to the specific country's context is required.

To ensure successful implementation, it is essential that the country's EC needs be documented, and acceptability established. A needs assessment can be understood as the systematic process of identifying unmet health-related needs of a population to facilitate adequate solutions [24]. It is a crucial step in successful implementation of health interventions as it informs an overall plan, helps identify targeted strategies and prioritise resources [25,26]. Acceptability is a multi-faceted construct that speaks to the appropriateness of a healthcare intervention [27] and must be considered when designing, implementing, and evaluating healthcare interventions [28,29].

This study aimed to assess the needs for, and acceptability of, the CFAR programme in the DRC.

## Methods

### Study design

Semi-structured interviews involving focus group discussions.

### Study setting

The study was conducted in Kinshasa, the capital city of the DRC, home to approximately 12 million people [30]. The DRC is located in Central Africa and has an estimated population around 100 million [31]. Its healthcare system is under resourced and many of its health indicators are concerning [32]. There is no formal national EMS system [20,22] nor adequate knowledge of first aid in households [33].

### Study population and sampling strategy

Through purposive sampling aiming to build representative groups of individuals capable of engaging in rich discussions to generate high-quality data, five focus groups were conducted (Table 1), each with eight participants (average of three women and five men, aged between 30 and 60 years).

**Table 1:** Overview of focus groups participants and selection process.

Focus groups	Participants selection
Group 1 (G1) Healthcare system cadres*	Participants were selected by the Head of the Planning Division of the National Directorate for Emergencies and Humanitarian Action (National Ministry of Health) among participants of the two-day WHO DRC Emergency Care System Assessment consensus meeting held in Kinshasa in 2020. Suggested participants lists were independently checked, amended, and approved by two research team members.
Group 2 (G2) **Frontline emergency care providers**⁎⁎	Participants were selected doctors and nurses who attended the above meeting, with the addition of the rare prehospital care providers from the fire brigade. The final list followed the same approval process as above.
Group 3 (G3) **Community health volunteers**⁎⁎⁎**(CHVs)**	Participants were selected by two research team members (4 for each) from a list of most active and available CHVs designated by the Chief Medical Officer of the health zone of Lemba in Kinshasa.
Group 4 (G4) **Community members** (Urban area)	Participants were lay members of the community of the urban health district of Mont-Ngafula selected by two research team members (4 for each) among respondents of a previous community-based survey on needs and supply of EC in Kinshasa
Group 5 (G5) **Community members** (Peri urban area)	Similar to the G4, but members were from the rural health district of Maluku 2 in Kinshasa

⁎ Healthcare workers (ambulance providers, nurses, or doctors) representing the first point of contact for patients needing emergency care.

⁎⁎ In the DRC healthcare system, group of laypersons with no medical background registered with the health zone, not on a payroll, and already involved in various health promotion programs such as family planning, malnutrition, childhood immunisation, malaria prevention, etc. They play a crucial role, bridging the health system and the community.

⁎⁎⁎ Healthcare system cadres: 2 senior members of the National Directorate for emergencies, 2 members of the Kinshasa provincial coordination for emergencies, 1 Chief Medical Officer of a health zone, 1 supervisor of CHVs at health zone level, 2 doctors from a General Referral Hospital.

### Data collection

Based on the study objectives and the groups profiles, four separate semi-structured facilitation guides were developed by the research team following iterative reviews and consensus. The guides included open ended questions relating to participants views on the current state of the country's EC system, CFAR suitability as an intervention, and the facilitators and barriers to its implementation and its sustainability. Two experienced facilitators led focus group discussions (FGDs) at neutral locations within a two-week period. Participants were briefly introduced to the concept of EC systems and given an overview of the CFAR programme. Discussions lasted approximately fifty minutes each and were conducted in French in G1 and G2, and in Lingala (vernacular) in the three other groups to enhance optimal participation. Interviews were audio recorded and transcribed. Another research team member fluent in both Lingala and French first went through the interviews transcripts to gain familiarity with the content before translation into English. Transcripts were verified by a different research team member. Anonymised transcripts were stored on a university-based cloud service (© OneDrive, Microsoft Corporation; Redmond, Washington, U.S.), with access restricted to the research team.

### Data analysis

Data analysis was led by a research team member who was not involved in any aspect of the data collection. De-identified transcripts were imported to NVivo version 14 (© QSR International, Melbourne, Australia) [34] and analysed using inductive-dominant content analysis in a five-step approach [35]: 1) immersion in the data, 2) extraction of meaning units, 3) condensing or summarising larger meaning units, 4) allocating codes to meaning units, 5) organising codes into categories. Open coding was used to systematically examine the data to identify initial labels that captured meaningful segments of information. The coding process was flexible and iterative, allowing for the refinement and development of codes as new insights emerged, and each transcript was reviewed at least twice. All the codes were checked by two research team members during the analysis process, with further discussion around any discrepancies, until consensus was reached. Following review of open coding, axial coding was used to establish relationships between the initial codes. This involved categorising codes into broader themes and subthemes, facilitating the identification of patterns within the data. Coding tree extracts were generated to visually depict the coding process; they were then compared with original transcripts to determine coherence, and another research team member verified the process.

### Trustworthiness

In keeping with Guba's constructs [36], qualitative rigour was ensured. Credibility was ensured by using well-established methods to collect data from consenting participants, debriefing sessions after FGD and the checking of translations and transcriptions [35]. Dependability was ensured through the detailed description of the methods that were employed for data collection and analysis, and through the reflective commentary related to prior assumptions and regular debriefings [35]. Confirmability was bolstered by regular debriefings and reflective commentary [35]. The use of the COREQ reporting checklist ensured transparency and further supported the dependability [35]. Though transferability of the results cannot be guaranteed, the detailed description of the setting and the sampled population will assist readers in gauging the generalisability of the findings. A pre-test of the data instrument was not performed beforehand to assess if it fits the purpose of the study.

### Ethics

Approvals were obtained from the University of Cape Town (HREC REF 088/2021) and the University of Kinshasa (REF ESP/CE/077/2021). Informed consent was obtained from all participants. Prior to commencing discussions, participants were informed of the purpose of the study, associated risks and benefits, and how confidentiality will be maintained. They were also told they could withdraw at any stage without any consequence.

## Results

Five FGD were conducted, with a total of 40 participants included. Discussions were structured around two main sections: the state of the EC in the DRC and the need for, and implementation of, a CFAR programme.

### 1) Status of emergency care in the DRC

**Fig. 1:**
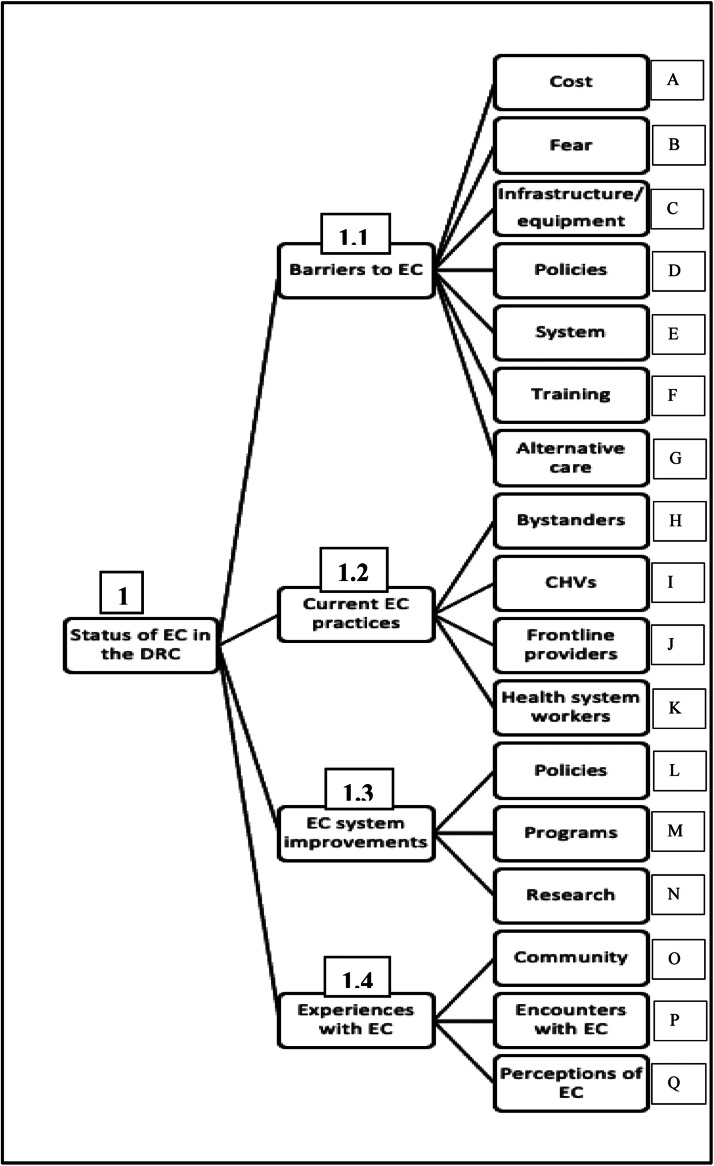
Coding Tree Extract – Status of Emergency Care in the DRC. (DRC: Democratic Republic of Congo / EC: Emergency Care / CHV: Community Health Volunteer / Aternative care: often called traditional medicine, including herbal treatments, naturopathy, homeophathy).

Four key categories were identified (Fig. 1). They broadly describe the DRC's EC context and enable a better understanding of the potential role of a layperson first responder system.

#### Barriers to EC

Substantial issues of planning, governance, and resources allocation were highlighted as obstacles that prevented a resilient and accessible EC system. Clinicians voiced a lack of trust in decision-makers as they felt that they were not investing enough and strategically in the strengthening of the country's healthcare system. (Reference: Fig 1, 1.1. D & E)

“*Politicians do not act as they should, everyone comes with their idea and sees their interests, so there are no favourable factors.” (G2)*

The lack of investment in EC was reported to hinder the availability of trained personnel and ambulances to transport patients to health care facilities. Many anecdotes of poor outcomes due to lack of prehospital care were provided. Even when patients made it to health facilities, care was often inadequate due to poor infrastructure and scarce equipment. (Reference: Fig 1, 1.1. C)

*“The situation is deteriorating; there are no adequate conditions for patients when they arrive, no equipment, loads of missing items, etc.” (G5)*

Cost was also a key driver of limited access, with community members and CHVs describing situations wherein care was delayed or not sought due to finances (Reference: Fig 1, 1.1. A)

*“They refused to attend to the patient at first because we had no money, and even when the money arrived, the care was still mediocre.” (G4)*

Participants also spoke of the despair and resignation of the mostly poor communities they lived in They were faced with a lack of functioning government facilities, which often led them to resort to mostly unaffordable private medical services. A combination of traditional beliefs, fear, and costs drove many to use over-the-counter medications, homemade solutions, and, very often, prayer. (Reference: Fig 1, 1.1. A, B & G)

*“Since we are jobless, we support ourselves with traditional medicines…” (G4)*

*“I called on my neighbours to help me to take her to the health centre after a nearby pastor prayed for her.” (G5)*

#### Current EC practices

Due to their proximity, CHVs were found to be already playing an inadvertent role in improving access to care. CHVs confirmed their interest in aiding in emergencies, especially with further first aid training (Reference: Fig 1, 1.2. H), with one stating:

*“During a routine community visit for polio vaccination for example, nothing would prevent me from assisting an injured person I come across; I cannot not intervene.”*

#### EC system improvements

Community members and CHVs described general discouragement about previous community-based programs, particularly lack of follow-up, additional training or equipment provision. The sustainability of small stipends that incentivise CHVs participation in various programmes was poor, which reduces participation in much-needed community interventions (Reference: Fig 1, 1.3. L).

*“…we were trained (as “community-based contraception sensitisers”) and were told that we will be receiving a certain amount of money monthly. But since our training to date, it's been almost 2 years, we haven't received anything. Now, when there is activity and we are needed, sometimes only 6 out of 11 health areas respond, the others don't come because promises made to them were not fulfilled.” (G3)*

An illustrative reference was made to a previous government attempt to develop a prehospital emergency care service in Kinshasa. (Reference: Fig 1, 1.3. M). Despite early success, the programme ultimately failed, due in large part to a lack of political support.

*“Between 2013–2016, we launched a pilot project of prehospital care service in Kinshasa (…) it barely lasted 6 months and stopped… the barrier for this project sustainability was the politicians…(they) did not follow, many vehicles broke down, staff training stopped halfway when we already had positive outcomes. The population accepted and supported, and many activities were carried out…” (G1)*

A participant also discussed the great potential of new leadership in the country to improve access to EC through the implementation of Universal Health Coverage (Reference: Fig 1, 1.3. L), noting:

*“This opportunity is the rise of the new leadership into power, with the vision of implementing universal health coverage…… A lot of lives will be saved……” (G2)*

#### Experiences with EC

Some participants described deeply personal encounters with the EC system, with some witnessing an emergency of someone they did not know and choose to intervene. (Reference: Fig 1, 1.4. P). Accessing and paying for EC were described as difficult, with one community member being forced to leverage their own home for payment:

*“I found my son (…) in severe pain; but we had no more money to go to the hospital. He previously had surgery but was still experiencing pain. The hospital managers had kept our property documents (as guarantee of payment), I didn't know what else to do, I had no money.” (G4)*

Perceptions of facility-based EC were mixed in and across participant groups. Some community members were satisfied with the care provided at hospitals, while others were discouraged, sharing stories of inadequate and delayed care. (Reference: Fig 1, 1.4. Q).

*“(…) there were no adequate conditions for patients when they arrive, no equipment, loads of missing items, etc.” (G5)*

### 2) The CFAR programme

**Fig. 2:**
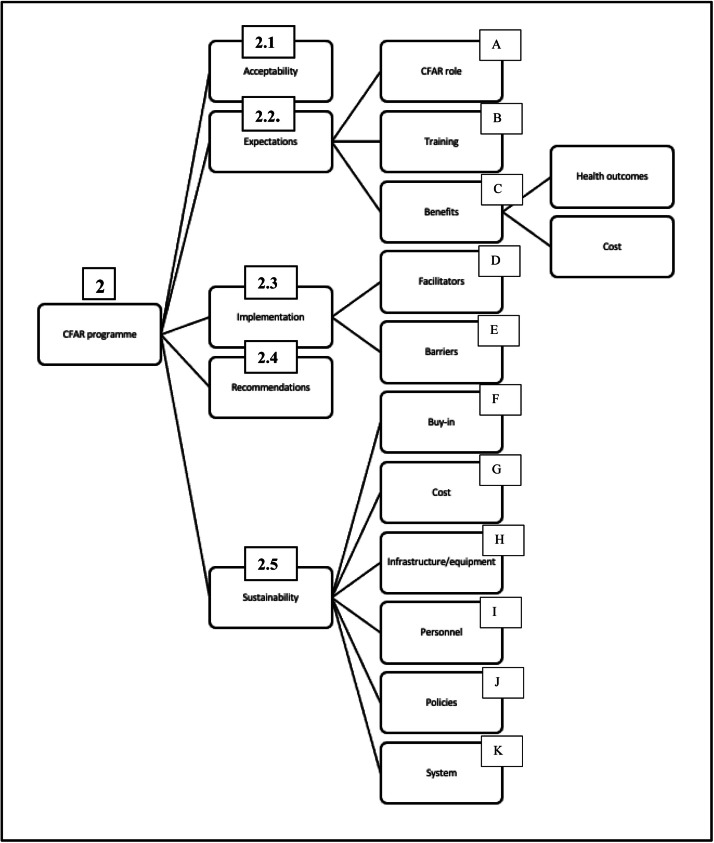
Coding Tree Extract – CFAR Programme.

Five categories pertaining to the need for, and sustainable implementation of, a CFAR programme were identified (Fig. 2).

#### Acceptability of a CFAR programme

**Fig. 3:**
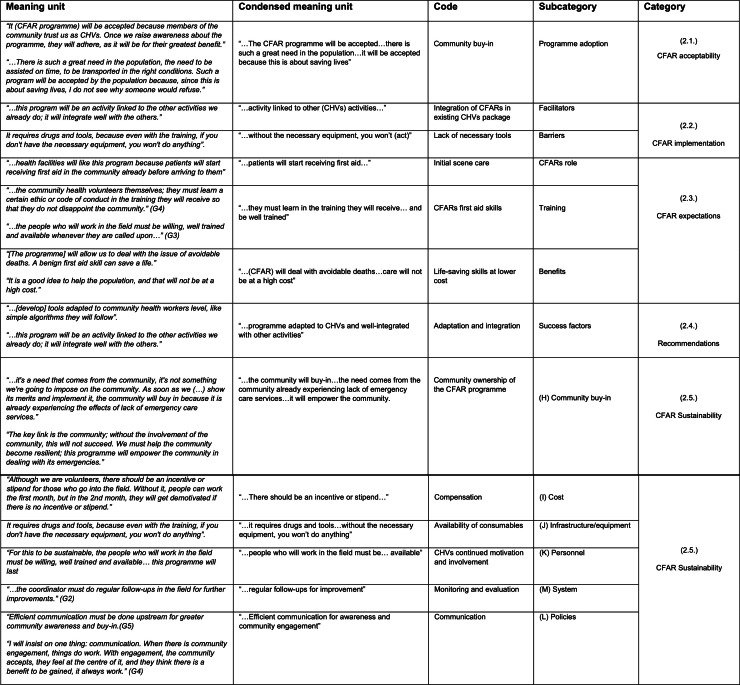
Categories.

CHVs were confident that the community would accept a CFAR programme, particularly because it would be implemented within the existing CHV intervention structure, which the community supports (Reference: Fig 2, 2.1.) (Fig. 3).

*“It will be accepted because members of the community trust us as CHVs. Once we raise awareness about the programme, they will adhere, as it will be for their greatest benefit.” (G3)*

Most clinicians were supportive and believed that a program that increased access to immediate care and transportation would improve health outcomes, and therefore be accepted in, and utilised by, the community. (Reference: Fig 2, 2.1.)

*“I say yes right away. There is such a great need in the population, the need to be assisted on time, to be transported in the right conditions. Such a program will be accepted by the population because, since this is about saving lives, I do not see why someone would refuse.” (G2)*

Health systems workers were generally supportive, but one did note that it would be essential to sensitise the community to the concept prior to implementation, or else “*we'd be putting the cart before the horse*.” A participant did note that, in order for acceptance to be widespread, there could not be costs associated with accessing care. (Reference: Fig 2, 2.1.).

*“It's a good thing, provided that the aim is to assist people in need and not to charge them and earn money.” (G4)*

#### Expectations for a CFAR programme

It was emphasised that the CFARs needed to be well trained to be competent, safe and of good ethical conduct (Reference: Fig 2, 2.2 B):

*“…the community health volunteers themselves; they must learn a certain ethic or code of conduct in the training they will receive so that they do not disappoint the community.” (G4)*

*“…the people who will work in the field must be willing, well trained and available whenever they are called upon…” (G3)*

They also argued that continuity with hospitals was requisite to a successful programme, so CFARs could smoothly handover patients. (Reference: Fig 2, 2.2 C).

*“If you make the commitment to train us, please also follow up on this, make all necessary items available in the health centres and hospitals, so that if we encounter cases, we can directly send them there.” (G3)*

Cost was a key driver of the enthusiasm about the programme, with the expectation that there would be little cost associated with accessing CFAR care: (Reference: Fig 2, 2.2 C).

*“It is a good idea to help the population, and that will not be at a high cost.”*

There was optimism that the programme could yield substantial health benefits, with frontline clinicians stressing on the positive impact on prehospital outcomes:

*“[The programme] will allow us to deal with the issue of avoidable deaths. A benign first aid skill can save a life.” (G2)*

#### Implementation of a CFAR programme

Potential barriers were elucidated, including longitudinal investment, with concerns that programme organisers may give up on implementation halfway through. This has occurred before, with previous health programs, and deeply frustrated the community. (Reference: Fig 2, 2.3 E).

*“(…) We gathered to give ideas, but until now, they have not returned.” (G3)*

Lack of community awareness regarding CFAR and lack of trust, both in the community and the health system, were also noted as barriers to the program's uptake. (Reference: Fig 2, 2.3 E). A CHV also highlighted the importance of proper identification in establishing trust with the community:

*“We who work on the ground, we know each other, in the neighbourhood, but we must first identify ourselves” (G3)*

Speaking from experience, a CHV noted that perception of CFARs lack of knowledge could reduce trust (Reference: Fig 2, 2.3 E).

*“If there is a need and people resort to you, and you cannot give a solution, then people will not have confidence, they will have doubts, and it will not work.” (G3)*

Finally, resources were perceived as a major barrier to the CFAR programme's success. Concerns about cost of supplies that would be necessary to provide appropriate care and CFARs were raised (Reference: Fig 2, 2.3 E):

*“It requires drugs and tools, because even with the training, if you don't have the necessary equipment, you won't do anything”.*

Despite several challenges, participants mostly maintained confidence that they could be overcome and highlighted several facilitators such as the integration of the program within the existing CHV structure. Being used to tasks being added to their repertoire of skills as new health needs arise, CHVs felt certain that the programme could be easily incorporated. (Reference: Fig 2, 2.3 D).

*“…this program will be an activity linked to the other activities we already do; it will integrate well with the others.” (G3)*

Health systems cadres felt that the community first-hand experience of the consequences of the absence of effective EC would lead to greater interest and buy-in for a CFAR programme. Finally, the potential benefits to healthcare facilities, including receiving patients that are better stabilised, would facilitate the programme's uptake (Reference: Fig 2, 2.3 E).

“…*health facilities will like this program because patients will start receiving first aid in the community already before arriving to them.”* (G3)

#### Recommendations for a CFAR programme

Participants insisted on the importance of having clear protocols for CHVs to follow (Reference: Fig 2, 2.4).

*“…[develop] tools adapted to community health workers level, like simple algorithms they will follow”.*

Regular evaluation and follow-up were recommended to regulate CFAR interventions and quantify impacts on the community. Community awareness of the CFAR program was advised, with suggestions for public advertisement through posters and community meetings. CHVs requested appropriate means of identification for CFARs to facilitate community recognition (Reference: Fig 2, 2.4):

*“(…) that we also have distinctive signs, so that I am identified when I am in a place where no one knows me.”*

#### Sustainability of a CFAR programme

Participants generally felt confident that the facilitators of the programme implementation would also impact its sustainability (Reference: Fig 2, 2.4):

*“I think it will be sustainable because it's a need that comes from the community, it's not something we're going to impose on the community. As soon as we (…) show its merits and implement it, the community will buy in because it is already experiencing the effects of lack of emergency care services.”*

Community buy-in and longitudinal engagement with citizens were thought to be of vital importance. Stories of failures of previous programmes due to lack of community acceptance were told (Reference: Fig 2, 2.5 F):

*“The key link is the community; without the involvement of the community, this will not succeed. We must help the community become resilient; this programme will empower the community in dealing with its emergencies.”*

However, concerns surrounding the sustainability of CFAR were raised. As CHVs are unpaid, adding an additional task to their workload may lead to burnout or take up too much time that they need to instead use to earn money. Several CHVs suggested compensation for their efforts, to improve motivation and financial sustainability (Reference: Fig 2, 2.5G):.

*“Although we are volunteers, there should be an incentive or stipend for those who go into the field. Without it, people can work the first month, but in the 2nd month, they will get demotivated if there is no incentive or stipend.” (G3)*

*“[A] small stipend for transport; it can vary between 5 and 10 $ per month over the three years.” (G3)*

Communication was also noted by participants as a key driver of long-term sustainability (Reference: Fig 2, 2.5 H):

*“Efficient communication must be done upstream for greater community awareness and buy-in.(G5)*

*“I will insist on one thing: communication. When there is community engagement, things do work. With engagement, the community accepts, they feel at the centre of it, and they think there is a benefit to be gained, it always work.” (G4)*

The importance of regulation of both training and programme administration was highlighted by G1 and G2 members, as well as quality assurance and ongoing programme assessment, to ensure that high quality care is being delivered (Reference: Fig 2, 2.5 I):

*“…the coordinator must do regular follow-ups in the field for further improvements.” (G2)*

Finally, continuity with the formal healthcare system was thought to be critical to the programme's success (Reference: Fig 2, 2.5 K):. Several participants called for integration of the CFAR with broader goals to improve facility-based care:

*“We could train community health workers, but if care in hospitals emergency rooms is not improved, it will be difficult … Therefore, the training of community health workers must go hand in hand with restructuring of the emergency care system.” (G2)*

## Discussion

The insights gained through the FGDs shed light on the current status of EC in the DRC and the potential for a CFAR programme to improve its OHEC delivery. Participants reported key challenges that align with findings of an evaluation conducted a decade ago [26], the gaps identified in a recent WHO-led EC system evaluation [24] and results of a household survey on the supply of EC in Kinshasa, DRC [25]. Barriers encompassed multiple aspects of health services delivery, including governance, financing, resource allocation, transportation, infrastructure, training, and cost.

Many participants conveyed eroded trust in policymakers and a lack of strategic investment in healthcare system strengthening. Lack of good governance and corruption have been found to undermine the delivery of essential healthcare services and result in poor health outcomes in LMICs [37]. Infrastructure and equipment were frequent barriers to community members successfully receiving EC services. The lack of a functioning ambulance service [24,26] was noted as a major impediment to access to early EC, as most patients have to walk or use private means to reach health facilities [25]. Similar to other African nations [38], conditions in emergency receiving areas of DRC hospitals were inadequate, with essential equipment and services often unavailable [26]. Lack of EC-specific training for healthcare providers was reiterated as a major hurdle to overcome [24,26]. In line with recommendations for the region [39], training at all levels would be essential to enhance the national system's capacity. CHVs could fill the gap in the meantime, receive first aid training and bridge the community and the out-of-hospital healthcare system [14]. The necessary funding to sustain the program over the long term and cost issues emerged as a significant barrier, with participants highlighting that the inability to pay upfront for EC services resulted in people not accessing care or reverting to self-medication [40]. These challenges underscore the importance of addressing financial barriers to ensure that individuals can access timely and quality EC when needed. WHO recommends universal health coverage [41] (UHC), and the country's political leadership has committed and started implementation.

CFAR as an intervention to strengthen out-of-hospital capacity in the DRC received positive reviews. Similar to other laypersons first responder programs [19,21,22], most participants showed support for the concept, with few reservations regarding its sustainability. Anticipated benefits included increased access, cost reduction, improved health outcomes, and prevention of unnecessary morbidity and mortality [42,43]. CFAR could also be a pillar for the development of resuscitation systems in LMIC settings with a growing burden of cardiovascular diseases and complications such as out-of-hospital cardiac arrests [44]. Acceptability was high across all groups, with only few participants expressing some hesitation. While community members were receptive to the concept and said they would utilise it, end-user education remains a key component of successful programme implementation [45]. The programme credibility was another ingredient for acceptability and to ensure uptake, the programme needs to be supported by those with subject matter expertise [46]. The healthcare providers endorsement could lend a sense of trust to the programme. Additionally, evidence of increased access to care following programmes involving CHVs [42,47] could increase credibility.

However, several potential barriers to implementation and sustainability were identified, including long-term investment and financing, as previous programmes were abandoned due to cost and lost interest frustrated the community. Additionally, should CFARs be unpaid as CHVs are, the longevity of the programme will not be guaranteed. Despite the CFAR intrinsic motivation to participate, without financial incentives, their good will might wane over time [48]. As highlighted, infrastructure and equipment may hinder the programme's success; longitudinal funding for expendable items will be vital.

Recommendations for a robust CFAR programme's execution and implementation in DRC were aligned with documented essential factors of successful community-based health interventions [49], including ensuring strong community buy-in, thoughtful participants selection, clear protocols, linkage to formal care through a basic communication and transportation system, and a framework for interaction with the healthcare system for greater integration. A greater chance of success was envisioned with CFAR programme integration into the existing CHV structure, as community members already trusted this effort and built a great trust over the years. Ongoing evaluation [50], another essential link in the implementation framework, was also recommended for continuous fine-tuning and improvement.

## Limitations

This study has a few limitations. Some participants may have not expressed their honest opinions but socially acceptable answers or refrained from expressing thoughts opposing the views of the majority, despite efforts made to ensure a conducive atmosphere for discussion. The FGD facilitators invited participants to freely express their views and assured them of anonymity. One of the facilitators is a Congolese healthcare provider who works within the system, and this could have biased his facilitation; this was countered by using a facilitation guide. Discussions were conducted in Lingala and French, then translated into English and transcribed. Though these translations and transcriptions were checked, a few misrepresentations could have happened as a few words do not necessarily translate. Finally, the inclusion of high-ranking Emergency Care Directorate leaders in one of the groups could have biased discussion as they seemed to dominate proceedings and speak longer, despite facilitators efforts to circumvent their influence.

## Conclusion

This study highlights barriers to resilient and accessible EC systems in the DRC, including planning and governance issues, inadequate resources, precarious financing, under-equipped health facilities, cost, and transportation challenges. Among the areas of the country's EC system requiring improvement, the initial response to emergencies in the community is likely one of the most fundamental. An adapted community-based first responder programme was deemed a useful and acceptable intervention to help increase out-of-hospital emergency care capacity in Kinshasa. The identified key potential facilitators and barriers to its implementation and sustainability can usefully guide the country's decisionmakers in their efforts to improve health outcomes from emergency conditions.

## Dissemination of results

A French translation of this article can be found as Supplementary file 2. The findings of this study will be collated into reports for the DRC Ministry of Health to help inform first responder programmes planning and development.

## Ethics approval

Approvals were obtained from the University of Cape Town (HREC REF 088/2021) and the University of Kinshasa's School of Public Health (REF ESP/CE/077/2021).

## Contributorship statement

K.D., E.M. and L.W. were involved in the conceptualisation, choice of the methodology, project administration and design of this study. K.D., E.M, J.Y. and L.W. were involved in the translation and contextualisation of the survey protocol. K.D., E.M.,J.P., J.Y.,C.C. and P.H. were involved in the acquisition, formal analysis or interpretation of data. K.D., J.P., J.Y., L.W., C.C. and P.H. were involved in drafting the manuscript, and all authors were involved in its review, editing and approval. L.W., C.C. and P.H. supervised the study.

## Funding

By the principal researcher.

## Declaration of competing interest

The authors have no conflict of interest to declare.
